# Re-Consenting Pediatric Research Participants as Legal Adulthood Approaches: Lessons from the SPARK Autism Study

**DOI:** 10.1007/s10803-022-05774-x

**Published:** 2022-11-02

**Authors:** Connie Anderson, Alan Iampieri, Leah Franklin, Amy Daniels, Katharine Diehl, J. Kiely Law

**Affiliations:** 1 Department of Health Sciences, Towson University, 8000 York Road - CHP Dean’s Office, Towson, MD 21252 USA; 2 Simons Foundation, 160 5th Avenue, New York, NY 10010 USA

**Keywords:** Adolescents, Autism spectrum disorders, Developmental disabilities, Informed consent, Longitudinal research, Qualitative research, Re-consenting, Transition to Adulthood

## Abstract

To explore issues surrounding re-consenting youth in longitudinal studies as they reach legal adulthood interviews were conducted with 46 parents plus 13 autistic teens enrolled in the Simons Foundation Powering Autism Research for Knowledge (SPARK) study. Qualitative analysis focused on family sensitivities regarding guardianship decisions, transition concerns, and the re-consenting process. Questions regarding guardianship were difficult for parents unsure of a teen’s future status. Mothers were key facilitators of re-consenting for soon-to-be-independent teens. As legal adulthood approached, parents were willing to assist teens with re-consenting but needed support, asking for multiple contacts, transition resources, and explanatory materials from the research team. Most teens were not cognizant of SPARK but willing to continue participation once made aware.

## Introduction

It is crucial to study conditions which arise in childhood, impact development, and persist into the adult years. Pediatric longitudinal research can answer key questions regarding course of symptom presentation, supports needed over time, and family impact as well as best practices in terms of treatment. It can also involve unique challenges. One of these is the issue of consent that arises when researchers intend to follow young participants into adulthood. First, they must determine if a young person is going to be placed under guardianship at the age of majority. If so, parental consent will endure. If instead the young person will become a legal adult, they must be re-consented as such. The current qualitative effort explores the complex issues that arise as encountered by the Simons Foundation Powering Autism Research for Knowledge (SPARK) study. These go beyond the logistical as the re-consenting process by its nature touches upon questions of a teen’s imagined future and degree of independence—emotionally charged issues for many families.

### A Word About Labels and Language

Debates about the most appropriate language to use when referring to autism are ongoing (Lei et al., 2021). For many years, “person-first” language was recommended, especially in professional settings. It was viewed as respectful to separate a disability from the one who “had” it: “person with autism.” Advocates disputed this view, describing autism as an essential part of themselves and not something negative from which they wished to be separated. They embraced “identity-first” language: “autistic person” (Kenny et al., 2016). When interacting with an individual, it is possible to use the terms they prefer, but that is not the case when writing about autism in the abstract. Authors must decide what language they will use. For the purposes of this paper, we are making a conscious choice to use “identity -first” language. We will also use the phrase “on the autism spectrum” – a descriptor that has been found to be acceptable among many autistic adults (Bury et al., 2020). Likewise, we will minimally use medicalized terms that are unavoidable where questions of diagnosis and epidemiology are concerned (e.g., “autism spectrum disorder” or “ASD”).

## Background

### Longitudinal Research Challenges and Re-Consenting

Researchers conducting longitudinal studies hope to keep their participants engaged over time, whether that is a year or a decade. A great deal of effort and expense go into recruitment and retention; maintaining a sample of sufficient size and diversity is important to validity of results and generalizability of findings. To recruit and keep study participants engaged, researchers craft careful communications, offer alternate modes of data collection (in-home vs. in-clinic interviews), and provide incentives like gift cards (Booker et al., 2011). They may brand their study with a recognizable logo (Taylor et al., 2015), and create community through a website or e-newsletter—what Teague et al. (2018) call “community building” (p. 10).

Additional effort is needed to recruit and retain participants who face exceptional daily struggles. For example, to maintain the participation of homeless people, researchers collect contact information of people with whom the homeless socialize, make community visits, and check in frequently (Hobden et al., 2011; Gerlitz et al., 2017). To accommodate people living in poverty—who may balance multiple jobs, struggle with childcare, and have little reliable transportation—researchers offer multiple data collection choices (online, in person, or by mail), allow for varied communication preferences (e.g., phone, text), provide a dedicated phone line for study assistance, and emphasize participant convenience (Nelson et al., 2021; Nicholson et al., 2011). Ethnic and racial minorities may feel mistrust towards research itself due to a long history of mistreatment by academic and clinical institutions. Researchers have learned to base recruitment in community settings with staff that are “culturally matched” to participants and an approach that builds on altruistic motives based in family and community (George et al., 2014, p. e25). In each case, researchers seek ways to reduce barriers and make participation easier.

Pediatric longitudinal studies and research registries face an additional dilemma. Participating children, whose consent was initially provided by parents, will reach legal adulthood at some point—a juncture we call “aging-up.” Unless an Institutional Review Board (IRB) waives the requirement for informed consent, researchers will need to re-consent these now young adult participants (Office of Human Research Protections, 2022).

In some cases, re-consent may not be required. A waiver may be granted even for ongoing analysis of data earlier collected from identifiable research subjects under certain circumstances, including those outlined in 45 CFR 46.116(f) (Code of Federal Regulations, 2022). These include a determination that the study involves minimal risk, could not be carried out without the requested waiver, and will not adversely impact the subjects. For example, the Pediatric Proton Consortium Registry, which follows children treated with proton radiotherapy via medical record review, was granted a waiver as long as at least three attempts were made to reach aging-up participants. Previously, more than 70% had failed to respond to attempts to reach and re-consent them. The waiver therefore made it possible to gain vital knowledge about young adult outcomes that would have otherwise been unattainable (Kassis et al., 2017). In the case of biobanks, Brothers & Wilfond (2017) argue that such waivers should be granted frequently. They assert that re-consenting young adults is “preferable but not obligatory” (p. 1) considering the balance that must be maintained between principles of informed consent and the fact that research using existing biological samples may enhance the public good.

Of course, pediatric studies that plan to keep their aging-up participants actively engaged in ongoing research have an obligation to re-consent them (Resnick, 2014). Unfortunately, there is a dearth of research on the longitudinal follow-up of pediatric research participants into adulthood. With the exception of highly specific studies like this one, few large, longitudinal research studies have grappled with reengaging a large child population as adults.

### Autism, SPARK, and the Need to Re-consent Aging-up Youth

Autism spectrum disorder (ASD), which currently effects 1 in 44 U.S. children (Maenner et al., 2021), is characterized by difficulty understanding and responding to the social world in a natural, intuitive fashion as well as restrictive, repetitive behaviors, activities, and interests (American Psychiatric Association, 2013). The term “spectrum” conveys the fact that people with this diagnosis vary greatly in intellectual capacity, language ability, social motivation, adaptive functioning, and many other traits. Indeed, it is due to this heterogeneity that extremely large samples are needed for autism research.

To meet this need, SPARK, supported by the Simons Foundation Autism Research Initiative (SFARI), has recruited over 100,000 children on the autism spectrum and their family members, as well as autistic adults, to participate in clinical, genetic, and survey research. While some SPARK participants are recruited from clinical sites around the United States and share medical records, the vast majority take part solely through online questionnaires. The data provided are of high quality. A recent study drew upon electronic medical records to confirm that caregiver- and self-reported ASD diagnoses in SPARK were accurate in 98% of cases (Fombonne et al., 2021).

Per the SPARK Consortium (2018), the project not only conducts its own research but enables the research of others through the Research Match program. External research projects are screened for quality and appropriateness and, if approved, receive assistance recruiting from among the thousands of SPARK participants who meet study criteria. Care is taken not to overwhelm any single participant with too many requests. The project’s overall aim is to accelerate autism research across the board.

Recruitment and retention are paramount. SPARK strives to maximize both by creating a sense of partnership with participants and making participation as convenient as possible. Through its website, newsletters, and webinars SPARK stays in touch, providing needed resources, sharing study results in family-friendly language, and otherwise striving to maintain a sense of community (The SPARK Consortium, 2018). Like other pediatric longitudinal studies, SPARK strives to be sensitive to the needs of participants who are caring for or have been affected by a condition that makes life challenging. Participants may be parents of young children navigating the special education system and advocating for treatments (Neely et al., 2012), or parents of teens worried about their child’s future services or employment (First et al., 2016). They may be adults on the spectrum with their own concerns, such as access to adequate vocational or mental health services (Schott et al., 2021).

SPARK faces many challenges in common with other pediatric longitudinal efforts, including one of particular urgency: how to engage and re-consent newly independent young adults so that they are not lost to this ongoing study. This is especially essential when it comes to autism. In 2018, only 3% of U.S. federal and private autism research funding was allocated to adult-focused projects (IACC, 2021). Lack of data makes it nearly impossible to accurately characterize autistic adults let alone inform data-driven policy (Robison, 2019). Therefore, the aim of the current effort is to provide recommendations to SPARK about how to best approach the re-consenting process. These recommendations involve identification of motivations in support of continued participation; types and timing of contact as adulthood approaches; and key parental concerns, sensitivities, and hopes that may influence the tone and wording of communications about a teen’s aging-up.

## Methods

This qualitative study is based on a grounded theory approach. This is an inductive method wherein researchers’ understanding of a phenomenon are permitted to evolve as data are collected, new insights emerge, and ever more pertinent questions are asked (Corbin & Strauss, 2014). In this case, what we sought was a more complete understanding of the circumstances and issues surrounding the transition to adulthood in general and re-consenting in particular.

To recruit families, our Towson University (TU) team coordinated with SPARK’s Research Match program. To begin, two TU Institutional Review Board (IRB) approvals were submitted to SPARK: one for the parent-focused study (*Parent and Youth Transition Hopes and Fears: Implications for Continued Engagement in SPARK, Part 1 - Parents)* and one for the teenager-focused study (*Part 2—Teens)*. In addition, a Researcher Distribution Agreement was executed so that TU could access limited SPARK data (e.g., teen gender, race) for any participant consented into the TU study.

Recruitment emails describing the TU effort and containing a link to “click here if interested in this study” were then sent to qualified families through the Research Match platform. Those who clicked were taken to an authorization screen. Once they provided authorization, SPARK could share their emails, phone numbers, and other limited data with the TU team. At that point, the TU researchers became the main point of contact, sending out detailed recruitment emails and scheduling interviews. Via an online form, parents provided consent for themselves and, when a teen agreed to their own interview, their teen. Participating teens completed an online assent form.

At intervals, the SPARK and TU teams gauged the diversity of the sample in terms of race, ethnicity, region, family income, and other factors. Representation by race and ethnicity was initially inadequate. The team then oversampled African-American and Hispanic families to address the representativeness of the study participants.

### Participants

To participate, a parent had to be enrolled with their family in SPARK and have a teenager on the autism spectrum aged 15–17.99. Over the course of several months, we recruited 46 parents of autistic teenagers from across the United States. In-depth interviews were conducted by CA, AI, and LF over phone or WebEx (an online virtual meeting system) according to the participant’s preference. Nearly 85% of parents preferred the phone over a video meeting.

Interviews lasted 20–60 min depending on participant engagement. Interviewers began by quickly reviewing some basic questions the parent had been provided in advance. These included items like “How old was your teenager when they received a first ASD diagnosis?” and “What type of high school does your teenager currently attend?” Next came a semi-structured interview. The interviewer followed the participant’s lead while covering a variety of topics, including a teen’s history, autism diagnosis, school experiences, and social life with particular emphasis on the approaching transition to adulthood. The current study is directed specifically at the interview’s final section with its focus on ongoing participation in autism research.

During parent interviews, it was possible to glean if the teen under discussion might be able to participate in their own separate interview. (Because these were phone or WebEx interviews, teens needed to be verbal and intellectually capable of answering some basic questions on their own.) If a teen appeared to be a possible interview candidate, parents were asked if they thought their teen might be interested. Some declined to reach out to their teen citing their likely disinterest or anxiety, while others agreed to discuss the possibility with their teen.

In all 22 teenagers were asked to participate, with 13 (59%) completing an interview: ten males, one female, and two identifying as nonbinary and preferring the pronouns “they/them.” Eight teens chose to be interviewed by phone and five via WebEx. In addition to experiences with and interest in autism research, interviews focused on family, school, friends, and the future. This last topic was probed only if a teen’s anxiety allowed as it has been reported that some teens on the autism spectrum find it difficult to contemplate the major changes the end of high school represents (Anderson et al., 2021; Cheak-Zamora et al., 2015). We stopped talking about the future if a teen changed the subject or outright told us it was something they preferred not to discuss. For example, one 15-year-old declared:

*I do not want to have to worry about adult stuff, I just want to not have to and just live in a cozy little cottage with a garden with snails in it… I hate having to worry about adult stuff that is so scary.*

Interviews usually began with a discussion of the teen’s interests which proved to be a valuable way to engage them. Drawing from the experience of Harrington et al. (2014), interviewers sought to match their manner to a teen’s needs, using simpler language when indicated, being concrete, and giving extra time to process if required. Some uttered only a few words in response to questions while others spoke at length. When a teen spoke very little, interviewers could not ascertain whether this was due to limited understanding, boredom, anxiety due to the unfamiliarity of the encounter, or autism-associated symptoms like difficulty with social reciprocity or social engagement.

Both parents and teens received a $50 Amazon gift card for sharing their insights with the research team.

The sample was diverse. Except for parent gender—only one father participated—some measure of diversity was achieved in race, ethnicity, parent education, family income, and region. (Table 1.) There was also diversity across the autism spectrum. All demographic and clinical details presented are based on self-report. We had no access to medical or educational records to verify diagnoses or other factors.

**Table 1:** Re-consenting “Aging-Up” Youth: Parent and Teen Characteristics

N	46
**Parent Characteristics**	
**Parents Interviewed**	
Mothers (%)	45 (98%)
**Interviewed by WebEx or Phone**	
Phone (%)	39 (85%)
**Mean Parent Age (SD)**	45 (6.1)
**U.S. Region** ^**a**^	
Northeast	11 (24%)
Southeast	11 (24%)
Midwest	10 (22%)
Pacific	9 (20%)
Southwest	4 (9%)
Rocky Mountains	1 (2%)
**Family Annual Income**	
Less than $25K (%)	5 (11%)
$25K-$49,999 (%)	7 (15%)
$50K-$74,999 (%)	10 (22%)
$75K-$99,999 (%)	6 (13%)
$100K-$149,999 (%)	9 (20%)
$150K or more (%)	8 (17%)
Refuse to answer (%)	1 (2%)
**Parent Education**	
High School or less (%)	2 (4%)
Some college or AA (%)	16 (35%)
Bachelor’s degree (%)	14 (30%)
Master’s degree (%)	11 (24%)
Doctoral/Professional degree (%)	3 (7%)
**Teen Characteristics**	
**Teen Gender** ^**a**^	
Male (%)	37 (80%)
Female (%)	7 (15%)
Nonbinary (%)	2 (4%)
**Teen Age** ^**a**^	
15 (%)	15 (33%)
16 (%)	26 (57%)
17 (%)	5 (11%)
**Teen Race**	
White (%)	28 (61%)
Black/African American (%)	7 (15%)
Multiracial (%)	5 (11%)
Asian (%)	2 (4%)
Other (%)	4 (9%)
**Teen Ethnicity**	
Hispanic/Latinx (%)	9 (20%)
**Age at ASD Diagnosis**	
0-3.99 years (%)	22 (48%)
4-7.99 years (%)	8 (17%)
8 years or older (%)	16 (35%)
**Guardianship Plans** ^**a**^	
Definitely Yes	15 (33%)
Definitely No	11 (24%)
Ambivalent	20 (44%)
^a^Do not add up to 100% due to rounding.	

### Analysis

The constant comparative method (Glaser, 1965; Boeije, 2002) associated with a grounded theory approach was employed to identify emergent themes and categories of experience reflected in participants’ narratives. These were to some extent informed by our earlier work with a similar population: young adults on the autism spectrum and their parents (Anderson & Butt, 2018).

We began with interview transcripts, then employed Atlas.ti qualitative analysis software to code portions of each narrative. Two coders independently marked each interview, meeting afterwards to merge their codes and discuss until agreeing on a final code. To enhance the study’s rigor, we “analyzed negative cases,” questioning emerging concepts when subsequent interviews failed to support them (Lincoln & Guba, 1985, p. 309). (For example, based on early interviews we expected parents’ guardianship plans to be unambiguous, falling into clear “yes” and “no” groups. We had to modify this conceptualization when subsequent interviewees described ambivalence regarding guardianship decisions.) For the current effort, we segregated material related most strongly to issues surrounding re-consenting including stressors at transition and attitudes about research participation.

## Results—Part 1: Emergent Groups “On the Spectrum”

To better grasp the experiences and views of these families and teens, we first divided them into meaningful groups based on a teenager’s place on the autism spectrum. Considering the many potential combinations of gifts and challenges embodied by any one autistic person, this was a complex task. We applied both deductive and inductive approaches. We had grappled with a similar question in an earlier study involving autistic young adults (Anderson & Butt, 2018) and kept these groupings in mind as we considered the new sample (deductive approach). However, we also applied the constant comparative method to look at the new sample through multiple lenses, with old categories existing as possibilities but not determinative. Through this inductive approach we winnowed from the teens’ characteristics two key categorization criteria: intellectual ability, which had also been a group criterion for our earlier sample, and psychiatric or behavioral issues.

Cognitive ability was conceptualized as a choice between “intellectually disabled (ID)” and “not intellectually disabled (NID).” We drew from three sources to determine whether a teen was designated ID or NID. First, there was the parent’s initial assessment of their teen’s cognitive ability at the time they signed up for SPARK. At that time, they were asked to mark down whether their child had Cognitive Impairment—true or false. Second, at the time of our interview we asked parents if their teen had co-occurring intellectual disability and noted their answer: ID—yes or no. If these answers contradicted, as they did in six cases, a review of the interview transcript was conducted by our team. ID or NID status was then assigned based on the parent’s description of their teen’s daily functioning, type of schools attended, high school document at completion (certificate vs. diploma) and expected age at graduation—typically 21 for those with ID and 18 for those without. In addition, there was one case where the parent told both SPARK and our interviewer that their teen was NID but the team designated the teen ID for purposes of group membership. This was based on several factors that taken all together seemed to indicate ID status: attending an ABA Therapy School into the teenage years, an expected age at high school graduation of 22, and being completely nonverbal.

Co-occurring psychiatric and/or behavioral issues were gleaned from parent accounts via questionnaires, which asked whether a teen had co-occurring anxiety, depression, or other psychiatric diagnoses, and interviews during which parents brought up challenges salient to them. These ranged from a teen’s tendency to shut down and cry under stress to a teen’s violent outbursts, suspension from school, suicidal ideation, or hospitalization. Based on the overall picture for any one individual, our team debated until consensus was reached on whether to categorize a teen’s psychiatric or behavioral issues as internalizing (with anxiety as a key feature; INT); externalizing (with aggression, outbursts, or self-injury; EXT); or “just autistic” with no particular psychiatric or behavioral problems reported. Externalizing behavior trumped internalizing behavior such that a person with both was placed in an externalizing category. The six groups that resulted from this exercise appear in Table 2 and are repeated here for the reader’s convenience: 4 (9%) ID only; 4 (9%) ID-INT; 2 (4%) ID-EXT; 7 (15%) NID only; 17 (37%) NID-INT; and 12 (26%) NID-EXT. The breakdown across intellectual ability was 10 (22%) ID and 36 (78%) NID. The breakdown across behavioral-psychiatric categories was 11 (24%) with none, 21 (46%) with internalizing issues, and 14 (30%) with externalizing issues.

**Table 2:** Groups on the Autism Spectrum (N = 46)

Group	n (%)	Description
ID	4 (9)	Autistic and ID; has no interfering psychiatric or behavioral issues
ID-INT	4 (9)	Autistic and ID; also has internalizing issues like anxiety
ID-EXT	2 (4)	Autistic and ID; also has externalizing behaviors like aggression, meltdowns, or self-injury
NID	7 (15)	Autistic without ID; has no interfering psychiatric or behavioral issues
NID-INT	17 (37)	Autistic without ID; also has internalizing issues like anxiety
NID-EXT	12 (26)	Autistic without ID; also has externalizing behaviors like aggression, meltdowns, or self-injury

Please note: to enhance readability and provide context, group type will follow teen pseudonyms throughout (e.g., “Suzie’s [NID-EXT] mother said…”).

It is our hope that this conceptualization of the autism spectrum will help us better understand the diverse experiences of our participants while avoiding the oversimplification of that spectrum into a “low- vs. high-functioning” binary. It is clear from the composition of these groups that there are some autistic ID individuals who have few “behavioral challenges” and might function better in the world than, for example, an autistic NID individual with frequent aggressive outbursts.

One theme linked to the low- vs. high-functioning binary came from just two families but was nevertheless significant. They expressed a belief that those who were “lower” are not of interest to researchers. Benjamin’s [ID] mother said:

*It’s not just teenagers that they haven’t really studied, but the lower functioning kids with autism sort of get ignored if you ask me… Especially when they get older. When they’re little, it’s more like, “Oh, let’s focus on it so we can fix them.” But then when they’re older, they’re like, “Oh, they’re not fixable.” They just kind of let them go... If I was going to say something about research that’s just what I would say. It’s like a black hole, nobody really goes there.*

## Results—Part 2: Views on Research and Re-consenting

Parents and autistic teens were able to steer the conversation to topics of importance to them through most of the interview. In the latter half, we would indicate it was time to focus on research and re-consenting by saying: “Now we’re going to switch gears and talk about autism research for a little bit.” Most participants followed our lead into a structured exchange around research participation and how best to engage aging-up teens. Once we were asking about re-consenting specifically, we found that it worked best to invite the parent to become a consultant. “What do you think would help parents understand this process? What subject line do you think would encourage parents to open an email about this topic?”

### Motivation to Participate in Research - Parents

To explore how likely parents were to either continue a dependent young adult’s participation in SPARK or to support a soon-to-be independent young adult’s re-consenting, we asked parents about their motivations for participating in research. Most parents (96%) could easily describe their reasons for involvement in both past and future autism research. The most frequently mentioned motivation was a need for answers. 57% hoped to understand autism better, and to have a clearer path forward. Some focused on their earliest experiences of autism and how a quest for answers still drives them. The mother of 16-year-old Charlie [NID-EXT] remarked:

*I’m sorry I get so choked up on this. That feeling of right after getting the diagnosis and going to all the things and still not having an answer… it was a long, long road to get through it and just not having answers. I am somebody who just needs logic to understand.*

Others sought answers about more current matters. Speaking of extreme behaviors emerging at puberty, Kevin’s [NID-EXT] mother said:

*I’m motivated by the fact that there needs to be more studies, there needs to be more research, there needs to be more generalities; research has got to start coming up with something other than research for the sake of research. That motivates me, so that other people don’t have to go through what I went through last year when we never saw it coming. I thought he was possessed. I really thought I needed to bring a priest in.*

As is captured in the phrase “so that other people don’t have to go through what I went through,” altruism was another driving factor, and one that was often mentioned alongside the need for answers. A desire to help other people on the spectrum and their families was in fact mentioned by 52% of parents. For example, Theo’s [NID-EXT] mother shared:

*I want to help other parents. I do not think the world understands how challenging being an autism mom or dad is… Even if it is just one person, even if I can just reach one person, or my son’s DNA helps change the life of one person, then I am willing to do that.*

About a third of parents specifically expressed a desire to learn more about the genetics of autism. This makes sense as SPARK is a study with a core genetic component and some families had contributed samples for DNA. Many, like Lily’s [NID-INT] mother, hoped for more information about any autism-associated genetics that might impact future children or grandchildren:

*I do have older children that are now starting to have children of their own. They’re wanting to know do they need to be concerned with their kids. Is it a genetic problem?*

Roughly 20% mentioned a quest for resources as motivating them to enroll in SPARK, making statements like “There’s literally zero resources available.” About the same number hoped researchers would focus on the needs of autistic adults to better inform and improve their support and success. The mother of one 17-year-old boy [ID] said:

*I’m living on the lower end of the spectrum with George. When we’re talking about the mid-to-lower end, what are we doing to make sure these adults have a quality of life and have a purpose, and meaning? What does that look like in the way of living arrangements, work arrangements, enrichment arrangements, so they can lead a balanced life?*

Only one parent mentioned money or incentives as motivating them to take part in SPARK or other autism research.

### Motivation to Participate in Research—Autistic Teens

Although some teens were able to discuss areas of passionate interest or share school experiences, many met questions about past or future research participation with “I don’t know.” Ten of the 13 did make at least one comment regarding research-oriented motivations, likes, dislikes, or fears. Of course, parents also remarked on their teen’s level of interest in research whether the teen had their own interview or not.

Parents viewed their teens’ motivations to participate in research as driven by altruism, a desire for incentives (e.g., gift cards), and/or an interest in science in general. Although only seven teens remarked upon their motivations for participating in research, their comments mostly matched the parents’ views. Regarding a desire for money or gift cards, 15-year-old Brandon [NID] declared:

*Basically, [a children’s hospital] said, “If you come do this, we’ll give you a gift card for...“ I think it was like $100 or something. And I was like, “I like money…!” I guarantee you gift cards will work.*

Altruism was also mentioned. 16-year-old Eric [NID-EXT] remarked:

*I just really want to help people, help the future deal with this. I mean that’s what they did with me. Because of the people years ago trying to understand autism and stuff like that, it’s helped me in my life with these scholarships and these classes that specifically help people like me. So I kind of want to repay that to society.*

Aspects of research that might discourage teen participation mentioned by parents and/or teens included physical procedures (e.g., blood draws), travel, having to be touched, and fear of being judged. Explained Elliott’s [NID-INT] mother:

*He was real suspicious at first like, “Why are we doing this? What is it? Are they going to be studying me, judging me?” sort of thing. And I explained that it was to help other people and that we might be able to learn stuff and help other people that have it… Then, he was like, “Oh, okay. Yeah, that’s cool.”*

### Re-consenting: Timing and Language

Parents asked about when and how often they should be approached regarding legal status at adulthood and the need for re-consenting used their teenager’s age as a marker. “Middle school” was the youngest age specified, and 18 was the oldest. It should be noted this is too late in most U.S. states as the matter usually must have been settled by the 18th birthday (Suh,^2020^). Many agreed that when teenagers were 15–16 years old was the ideal time to begin informing parents that they would soon be asked about guardianship and re-consenting. Lily’s [NID-INT] mother was typical:

*I would say between 15-to-16. I mean, parents that stay on top of their kid like I do, you’re able to see what your child’s able to do now… I would say the latest, 16.*

Parents also shared their views about the best terms to use when reaching out about re-consenting. Above all, they urged researchers to keep language around these topics as simple as possible. They emphasized that parents taking in this information have disparate educational backgrounds and, depending on their teen’s situation, may also be coping with high levels of stress. Theo’s [NID-EXT] mother said:

*I would say for most parents, just keep it as simple as possible… like you would if you were talking to someone off the street. It gets really overwhelming… The amount of phone calls that I have to make, the emails, the phone calls, the doctor appointments, and the IEPs… It gets very overwhelming as a mom.*

Jesse’s [NID-INT] mother suggested getting not just parents, but teens involved early so that reaching out about re-consenting would be part of regular, expected contact:

*Hopefully, if they’ve been doing it all through high school, they already feel connected to the study… It might be something that they’re already invested in and feel is something that is part of their routine.*

We asked how researchers should broach the topic of guardianship and re-consenting. We learned that the former was an anxiety-producing topic for many, with implications for email subject lines and other communications. The emotional impact and salience of the term “guardianship” varied a great deal, often depending on where a teen was on the spectrum. For the 33% of parents who had long anticipated becoming their teen’s guardian, there was a sense of acceptance and clarity. Said Jack’s [ID-EXT] mother:

*I definitely have to apply for guardianship… He’s obviously not capable of making his own decisions… The guardianship thing is very, very important.*

Similarly, the 24% who were certain they would not arrange for guardianship dismissed the term out of hand. It was simply a concept that did not apply to them. Peter’s [NID-INT] mother shared:

*I have not thought about that at all–and I guess maybe the answer is that it’s something I haven’t considered because maybe I don’t think it’s necessary to do that for him.*

For the 44% who were unsure or ambivalent, however, “guardianship” was an uncomfortable term, eliciting worry and sometimes sadness. Julian’s [ID-INT] mother explained:

*For me, it’s going to be a difficult thing. The reason why is because when you have this little baby in the world your goal is for him to grow up and become a doctor or whatever you want him to be. Once you see this point, then some of those things are over. It’s almost a grieving pattern.*

When it came to the language used to communicate with families about these matters, most parents preferred the word “transition” to “guardianship” for use in email subject lines or other outreach. It was broader, encompassing the multiplicity of concerns families face as their teens approach the end of high school. It was forward-looking without the triggers or potential dismissal (“this doesn’t apply to us”) associated with the term “guardianship.” Said Kyle’s [NID-EXT] mother:

*Guardianship I’ve heard about just through his school meetings… but “transitioning into adulthood” would be something I’d be like, “That’s something that we’re about to go through, so I’d really like information on that.” That’s something that would catch my eye.*

### Brainstorming the Re-consenting Process

Parents were asked to share their ideas regarding the best way for SPARK to engage both teens and parents in the re-consenting process. For about-to-be-legal-adults, how should they reach out to encourage completion of a new online consent? What role, if any, could parents play?

There was no consensus regarding the best mode to use to contact aging-up teens. Whether email, texting, phone, or social media was a preferred method of communication for a teen was specific to the individual with no overall theme emerging.

A theme that did surface was that of mothers as facilitators of continued “independent adult” participation in SPARK. Perhaps unsurprisingly, teens were described as focused on their own concerns and unlikely to wade through a lengthy email or mail packet without some action by their parent. Therefore, some parents asked for materials to help them explain re-consenting to their teen—most often a video (perhaps on YouTube) or handout. Preferences differed. “If it’s a video, he’ll watch it in a hot second,” said one parent. Remarked another, “A handout would be more helpful…I think with a video, he’s probably going to tune it out.”

Either way, parents were clear that the best way to insure their teen saw and acted upon re-consenting communications was to keep parents—most often mothers—alert and involved. One way to do that was to send the teen-intended packet via mail. Charlie’s [NID-EXT] mother stated:

*The only thing I could say at this time is if mail was addressed and I saw the mail and it said it’s from SPARK, and if it is addressed to my son, I could pull it out of the pile and say, “Okay, let’s open this together and see what’s in there…” But sending him something via email or text, I would have no idea.*

The teens were described as perhaps interested, but busy, and sometimes disorganized, with a tendency not to see emails and to lose materials. This may reflect the executive-functioning challenges described in programs aimed at addressing autistic characteristics when planning school-based interventions (Aspy & Grossman, 2011). Some mothers explained that they and their teens had developed a system where the mother monitored deadlines and major events to help keep their teen on track with reminders or a little bit of assistance. The more the re-consenting process would permit that system to work, the more it would succeed. Tim’s [NID-INT] mother explained:

*He’s very open to my suggestions and he knows that if he asks why, we’re going to sit and we’re going to have a discussion as to the pros and cons of it. If I tell him, hey, you need to continue on with this program because in the long run it’s going to help you help somebody else, he’ll probably say, “Yeah.” And then he’ll probably say, “Hey, you need to help me with this.”*

Said Eric’s [NID-EXT] mother:

*As long as there was an explanation I could attach to it, he’d be willing. I know he’s going to be an adult, but he has a pretty open relationship with me… He’d most likely call me and be like, “Mom, you signed me up for that SPARK thing. What does this mean?“ That’s literally what he would say.*

Parents also mentioned that resources around the transition to adulthood were desperately needed. As mentioned in the section on research motivation, this was part of why some had joined SPARK in the first place. Parents felt that as children aged there were fewer concrete resources and that most were disorganized and unhelpful, whether provided by the school, adult service agencies, or advocacy organizations. Ava’s [NID-INT] mother said:

*I’d say that transition period in adulthood, there’s not a lot out there. I feel like the older they are, the less support there is.*

Part of that transition period involves becoming familiar with and—if they apply to you—making decisions about guardianship, power of attorney, special needs trusts, Supplemental Security Income (SSI; benefits for low-income people who are blind or disabled), Medicaid, Vocational Rehabilitation, state programs for adults with developmental disabilities, and postsecondary education programs accommodating students on the autism spectrum. Parents thought it would be ideal if SPARK made several contacts with them through their teenager’s 16th and 17th years. (This would be the last two years of high school for many NID teens.) Offering resources around these complex and worry-inducing topics might help parents stay engaged through the teen re-consenting process. The initial contacts might outline some of the upcoming decision points and likely places to find out more regarding a state’s services or laws. Such assistance, said Richard’s [NID-INT] mother:

*…would be very helpful because for me I’m hearing that and thinking about it and going crap, which way am I going?*

As Ava’s [NID-INT] mother put it, if SPARK provided some of this information just when it was required, parents might very naturally stay engaged and facilitate their aging-up teenager’s re-consent:

*I think it’d be great to say, “Hey, these are some things that some parents might be thinking about” and maybe even directing them to resources... So, you’re kind of giving them information but you’re getting information from them at the same time because I think not everyone knows to go look for those resources.*

As age 18 approached, communications could switch to when and how parents would be asked about guardianship decisions and re-consenting, and when to expect aging-up teens to receive their own communication or packet of information from SPARK.

## Discussion

Interviews with 46 parents of autistic teenagers provided insight into how to keep them and their “aging-up” adolescents involved in a longitudinal autism research study. Interviews with 13 of the teenagers added an understanding of their concerns and motivations regarding research participation. A summary of resulting recommendations is provided in Table 3.

**Table 3:** Summary of Re-consenting Recommendations

Topic	Recommendation
Timing and frequency of re-consenting contacts	Parents recommend age 15–16 as the best time to begin twice yearly contact about re-consenting – increasing in frequency during the 17th year immediately prior to legal adulthood (age 18 in most states).
Communication about re-consenting	Parents recommend keeping language simple; don’t assume people know what “re-consenting” is or the legal implications of reaching the age of majority for research participation. Email subject line “transition to adulthood” viewed as less threatening than “legal status” or “guardianship,” and is better understood than “re-consenting.”
Parent as possible facilitator of teen re-consenting and ongoing participation	Consider collaborating with parent to engage and inform soon-to-be independent teens about the opportunity to continue in a study and the need to re-consent; consider mailing packet so parent and teen are aware of upcoming deadlines. (Emails/texts sent to teen alone may go unnoticed by soon-to-be-independent teens—or not seem relevant—and never reach parent’s awareness.) Consider including permission for parents to be copied on important study emails or invitations in re-consent document as long as an “opt out” is offered.
Re-consenting communications: Parents	Pair re-consenting reminders with critically needed information such as forewarning of key decision points in the transition process, legal status (and options) upon the age of majority, timing of high school exit, resources for college, employment, or adult services. Focus more on re-consenting process itself as deadline approaches. Provide parental guidelines that encourage reminders or helpful explanations for teens but discourage exertion of undue pressure to participate.
Re-consenting communications: Teens	Make contact with the teen at age 16 so communications from the project is part of regular, expected contact. Provide parents with hand-outs and links to videos that will help them explain re-consenting to their teen; prepare teen-directed materials for teens not just about re-consenting but with a focus on their areas of concern (e.g., relationships, college, employment).
Parent research motivations	Parents’ top motivations for research participation included a quest for answers, altruism, an interest in family genetics, an expectation of helpful resources, and hope for help leading to their teen’s adult support or success. Providing needed, high-quality resources about the transition to adulthood, guardianship (and alternatives), adult services, employment, and post-secondary education along with requests for action is key. Gift cards or other incentives may not be necessary.
Teen research motivations	Teens’ top motivations for research participation included altruism and incentives. Gift cards or other incentives may be helpful.
Parents of ID teens may believe researchers are not interested in them or their teen	Parents of intellectually disabled teens may not believe their information is of interest to researchers Autism researchers should be concrete and direct about their desire to include families of ID teens. Mentions of the “autism spectrum” may not be enough to reassure these families that they are wanted and welcome.
Parental sensitivity to guardianship topic difficult to predict	No matter a teen’s cognitive or behavioral profile, families may find the decision surrounding guardianship emotionally difficult. Communications should be crafted to sensitively broach the topic in case a parent finds this topic challenging.
Families are not always aware of alternatives to guardianship or how legal status impacts continuing participation in a research project	Materials should explain alternatives to guardianship from powers-of-attorney to Supported Decision Making (SDM) models; many families are eager for more information about these matters and also need assistance understanding how they relate to their teen’s ability to continue participating in a research project they joined as a child with their parent’s consent.

### Issues Related to the Heterogeneity of the Autism Spectrum

Immediately apparent even in this small sample was the diversity of individuals bearing the autism label. We listened to families relate their teen’s varied characteristics, the impact of these on their family, and related triumphs and difficulties. Dissatisfied with the frequent division of autistic individuals into a “lower- vs. higher-functioning” dichotomy, we employed the constant comparative method to identify the simplest and most germane subgroups. As we did so, we benefitted from having performed a similar exercise on an earlier sample of autistic young adults –a deductive aspect of an otherwise inductive process. We identified six groups based on just two traits: cognitive ability (ID vs. NID) and an assessment of behavioral-psychiatric issues (internalizing, externalizing, none). With a small number of survey questions, researchers could similarly divide individuals on the autism spectrum into groups like these, permitting them to investigate challenges, design communications, and offer resources matched to a group’s specific profile.

One topic broached by two mothers of ID teens was that “low-functioning” individuals are not of interest to researchers. It may be that this is something families with ID and/or nonverbal children have internalized over time. McKinney et al., (2021) note that families of autistic children with “profound communication difficulties” have often been excluded from research and “may become increasingly hopeless and isolated” (p. 1636). Autism researchers should be concrete and direct about their desire to include such families. Mentions of the “autism spectrum” like what appeared in our research invitation may not be enough to reassure these families that they are wanted and welcome.

A critical step in the re-consenting process is determining intended guardianship status. Will a teen become a legal adult at the age of majority and need to be re-consented or not? Speaking with families, we found this was not as straightforward as might be expected. First, although parents of ID teens are more likely than those of NID teens to have definite guardianship plans (70% vs. 22%), there were exceptions. Three (30%) of the parents of ID teens were ambivalent, with two of these describing the guardianship decision as an emotional struggle. Likewise, a teen’s behavioral profile did not always correspond with guardianship intentions. For example, guardianship was definite for only half of teens with externalizing behaviors, including aggression. Most important to note, the twenty (43%) parents expressing ambivalence found the guardianship question distressing no matter their teen’s IQ or behaviors. As there is no easy way to predict an individual parent’s feelings related to guardianship, communication around guardianship decisions requires special sensitivity.

It also requires awareness of current debates regarding guardianship, something that seemed unfamiliar to nearly all parent participants. There has been a philosophical shift towards less restrictive arrangements for adults who may need assistance making decisions (Blanck, 2021), including increasing interest in a supported decision-making (SDM) model wherein people with limitations seek out help making decisions from family and friends just as typical people do, whether with a formalized arrangement in place or not (Kohn, 2021). This reflects “a change of paradigm in the conception of disability from a medical capacity/incapacity model to a social model based on supports and founded on human rights” as set forth in the United Nations Convention on the Rights of Persons with Disabilities (Gómez Penalver et al., 2021, p. 230). Courts have begun to incorporate these principles, with 22% of 51 U.S. jurisdictions establishing laws that recognize SDM and a majority at least requiring consideration of less restrictive alternatives to full guardianship (Martinis et al., 2021). Unfortunately, schools’ habitual transition practices tend to bias parents in the direction of full guardianship resulting in what the National Council on Disability (2019) calls the “school-to-guardianship pipeline” (p. 29). This may explain why more than half of adults with autism using developmental disability services are under full guardianship (Roux et al., 2021). Indeed, Raley et al., (2020) have called for an amendment to the Individuals with Disabilities Education Act (IDEA) so that parents are provided information about less drastic alternatives including narrowly focused powers of attorney (e.g., medical, financial) and SDM models that preserve a young adult’s right to self-determination. Such alternatives should be of particular interest to families of teens with autism or any other condition with a wide spectrum of functioning. It is interesting to note that even when prompted, only three parents participating in the current study mentioned the possibility of a power of attorney for their teen and only one mentioned the concept of a “limited guardianship.” None used the term “supported decision-making.” Understanding alternatives to guardianship is not only an area where families apparently need support, but one researchers and IRBs will also need to understand better as they are growing in popularity and have implications for consent.

### Motivation for Participating in Research

Motivations for taking part in research were somewhat different for parents and teens. Parents’ top reasons for participating in SPARK were a quest for answers and altruism followed by an interest in family genetics, an expectation of helpful resources, and hope for help that would lead to their teen’s adult support or success. Only one mentioned her interest in receiving a gift card or other incentive. Of 27 parents who commented on their teen’s motivations for research participation, about half mentioned their interest in incentives while 41% mentioned altruism. Although only seven teens discussed motivation regarding research participation, these were also their most frequently mentioned motivations.

Based on these findings, to recruit or retain parents of autistic teens it may be most helpful to emphasize the answers—including genetic ones—researchers hope to generate; the greater good they hope to serve; and the useful information that will be provided along the way. This last is particularly significant as these teens approach the transition to adulthood. Resources about special education and childhood concerns are plentiful. Those about adult paths, services, and outcomes are not. SPARK already provides family-friendly articles, e-newsletters, and webinars to their participants –innovative approaches that have been associated with improved retention rates (Teague et al., 2018). Offering more of the desperately needed information the parents of aging-up teens require may help keep them connected to SPARK through the re-consenting process. This would adhere to what Vincent et al. (2012) call “customer service principles” (p. 70), namely giving participants with every contact a positive experience and sense that the study team understands and sincerely cares about their needs. It would likewise tailor retention strategies to this unique group of participants–a practice likewise associated with high retention rates in longitudinal studies (Abshire et al., 2017). (For a more detailed discussion of the desired resources, see the section below.)

### Reaching Out to Re-consent

A key goal of this effort was to determine how best to reach out to SPARK families with an aging-up teen both to inquire about guardianship and to broach re-consenting for those about to become legal adults. When exploring these issues, we found it most effective to enter a collaborative mode with these families, taking the time to explain these complex concepts and then inviting parental input. On the whole, parents recommended beginning contact about guardianship and re-consenting as the teen became 15–16 years old. This would allow for multiple contacts—perhaps one or two a year—until the 17th birthday when contacts could become more frequent.

Parents suggested that any reminders be paired with critically needed information. Resources were viewed as of high value and something that would continue to engage them. Specifically, parents were looking for forewarning of key decision points in the transition process. What should they be thinking about in terms of their teen’s eventual legal status, high school program (should they exit at 18 or 21?), postsecondary education, employment, or adult services? General information would be welcome, as would links to more specific information by state. For example, parents might benefit from learning about vocational rehabilitation (VR) agencies and the services they provide. They would also benefit from being directed to the specific agency, of whatever name and constitution, in their own state. One of the impediments to navigating adult services is that each state has completely different agencies and acronyms. In Maryland, for example, the Division of Rehabilitation Services (DORS) is the VR agency which not only retrains people who become disabled so they can return to employment but provides a community college support program for degree-seeking autistic students (DORS, n.d. *a*) as well as a driver’s training program for autistic individuals (DORS, n.d. *b*).

It was proposed that the nature of content morph over time, from general transition information in the early high school years to more fine-tuned instructions about re-consenting as age 18 approached. Along with information for parents, some suggested, it would be wise to provide materials they could use to explain the consent process to their teen. Engaging, easy-to-understand hand-outs or videos (perhaps via YouTube links) were suggested.

Also critical was parents’ view of the role they would likely play in any re-consenting process. Mothers sometimes explained that they had developed methods of helping keep their teen on track with regards to homework and other activities and could play this same role vis a vis re-consenting. To do so they needed to know when the re-consenting moment was coming. If an email or text was sent to their teen and missed or ignored, they would be unable to offer assistance or reminders. A large packet coming by mail they would see; otherwise, they needed some kind of notification that the teen was about to be contacted.

This raises another matter—that of ongoing parental involvement, at least insofar as knowing a communication is coming and being ready to remind a now young adult that the parent is happy to assist with any action needed. For instance, the re-consent completed by new adults might be crafted to include permission for parents to be copied on important study emails or invitations as long as the adult is given the opportunity to “opt out” of such an arrangement. Perhaps there would also need to be some parental guidelines that encourage reminders or helpful explanations but discourage exertion of undue pressure to participate. Indeed, earlier communications might touch on alternatives to guardianship and themes of self-determination that prepare parents to consider their role in this light.

Those taking part in the current study urged researchers to use plain language and make it easy for overwhelmed parents to absorb and respond to information. Parents also recommended using email subject lines and other communications with the term “transition to adulthood.” This was viewed as positive in a way that “legal status” or “guardianship” was not. (Recall that the term “guardianship” elicited dismissal from some and discomfort from others.) Regarding the form contact with teens should take there was no agreement. Mail, phone calls, texts, email, and social media were all mentioned, both positively and negatively. The conclusion that can be drawn is that, in order to reach every teen, multiple modes of communication would be best.

### Limitations and Strengths

A limitation of the current study is that it is based on a small convenience sample of 46 families. (The fact that they were all previously enrolled in the SPARK study is not a limitation as the question of re-consenting only applies to families taking part in ongoing research.) In addition, only 10 of 13 participating teens shared thoughts about research participation, limiting our understanding of their views. Finally, ASD and other diagnoses were based on parent and/or teen report rather than clinical or educational records, although a recent study comparing the parent reported ASD diagnosis in the SPARK study to medical record review demonstrated high validity (Fombonne et al., 2021). Strengths of the study include representation of families of autistic teenagers from across the United States who were racially, ethnically, and socio-economically diverse. Also captured was diversity across the autism spectrum both in terms of intellectual capacity and behavioral or psychiatric challenges. Although not all teenagers could be interviewed, thirteen were. Participants were able to direct interviews towards the topics of most importance to them, permitting phenomena not previously contemplated by the research team to be considered (e.g., attitudes towards guardianship and their impact on the re-consenting process).

## Conclusion

Pediatric longitudinal studies seeking to re-consent teen participants as they become legal adults may encounter unforeseen obstacles. When a sample includes teens who may not have full rights as adults, these include family sensitivities around guardianship decisions critical to young participants’ status at the age of majority. Such decisions have a direct effect on researchers who must be able to divide participants approaching adulthood into those who do and do not need to be re-consented. Families whose teen will become an independent adult may need support understanding re-consenting as well as materials that will help them explain the ongoing study and consent process to their teen. Additional research is needed to further explore these dynamics across different types of studies with different populations of interest. Also critical for longitudinal research is an investigation of the growing impact of alternatives to guardianship and the implications of these for young adults’ legal status and capacity to re-consent.
